# Supplementary figures and images for: The activation cascade of the broad-spectrum antiviral bemnifosbuvir characterized at atomic resolution
Source: PLoS Biol. 2024 Aug 27;22(8):e3002743. doi: 10.1371/journal.pbio.3002743 (PMC11349198; doi:10.1371/journal.pbio.3002743)

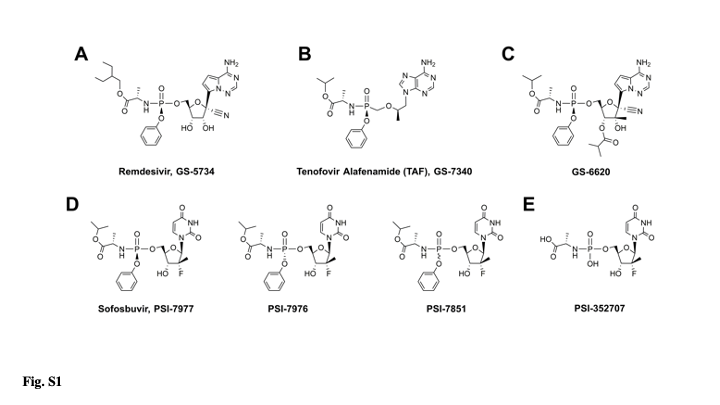

Supplement: S1 Fig — (A) RDV or GS-5734, aryloxy phophoramidate prodrug of an adenosine analogue, with 1′-C-nucleoside bond and 1′-cyano substitution. (B) TAF or GS-7340, prodrug of tenofovir, acyclic phosphonate analogue of adenosine monophosphate. (C) GS-6620, aryloxy phosphoramidate prodrug of an adenosine analogue, with 1′-C-nucleoside bond and 1′-cyano-2′-C-methyl substitutions. (D) SOF or PSI-7977 (SP diastereoisomer) and related compounds PSI-7976 (RP diastereoisomer) and PSI-7851 (mixture of both diastereoisomers), aryloxy phosphoramidate prodrug of uridine analogue with a 2′-fluoro-2′ -C-methyl modified ribose. (E) PSI-352707, phosphoramidate metabolite of SOF. NA, nucleoside/nucleotide analogue; RDV, remdesivir; SOF, sofosbuvir; TAF, tenofovir alafenamide. (TIFF) [file pbio.3002743.s001.tiff]

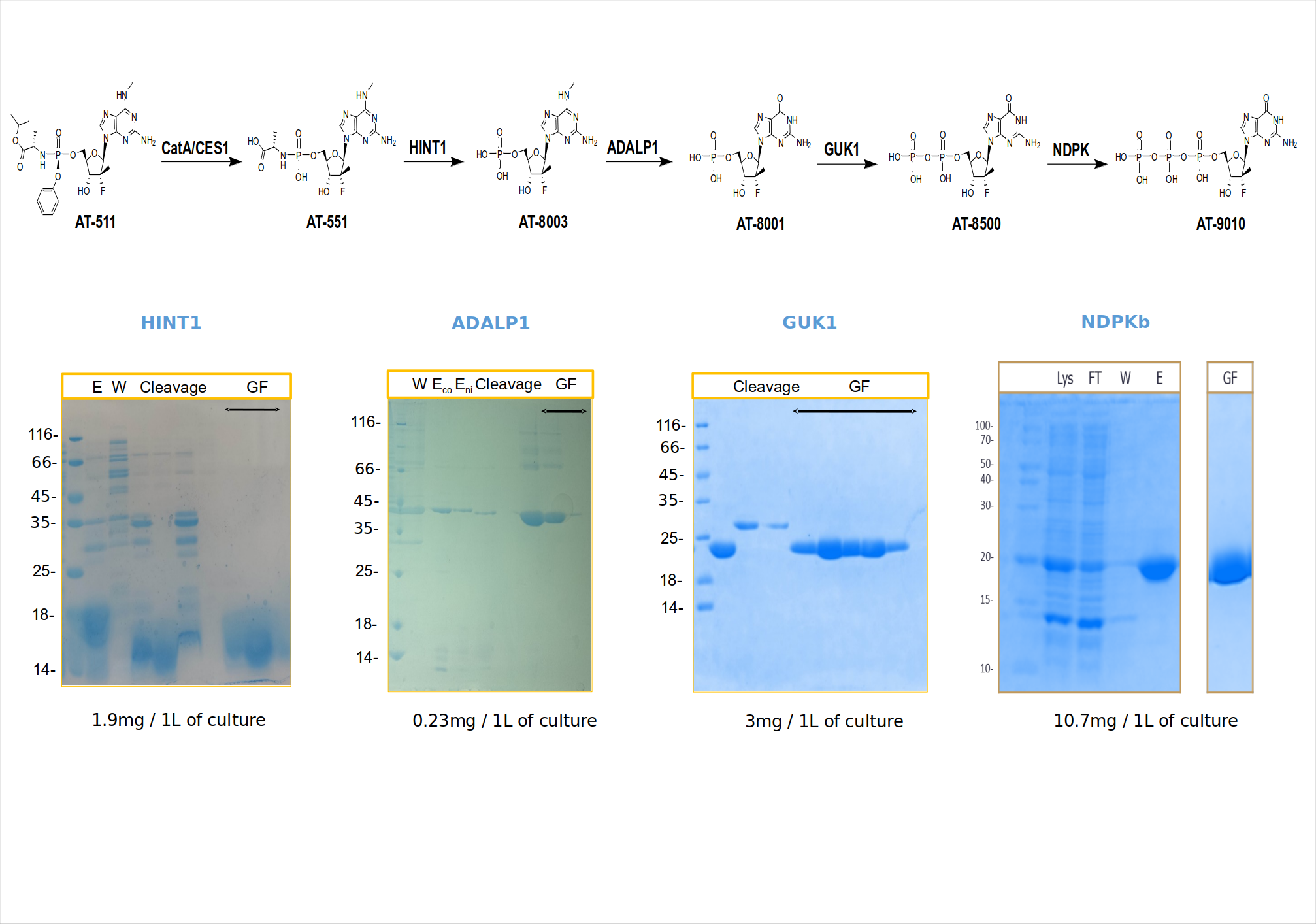

Supplement: S2 Fig — Key: Lysate (Lys), Flow-through (FT), Wash (W), Eluate (E), Tag cleavage eluate (Cleavage), Gel filtration (GF). The data underlying this supporting figure can be found at https://zenodo.org/records/12606239. ADALP1, adenosine deaminase-like protein 1; GUK1, guanylate kinase 1; HINT1, histidine triad nucleotide 1; NDPK, nucleoside diphosphate kinase. (TIF) [file pbio.3002743.s002.tif]

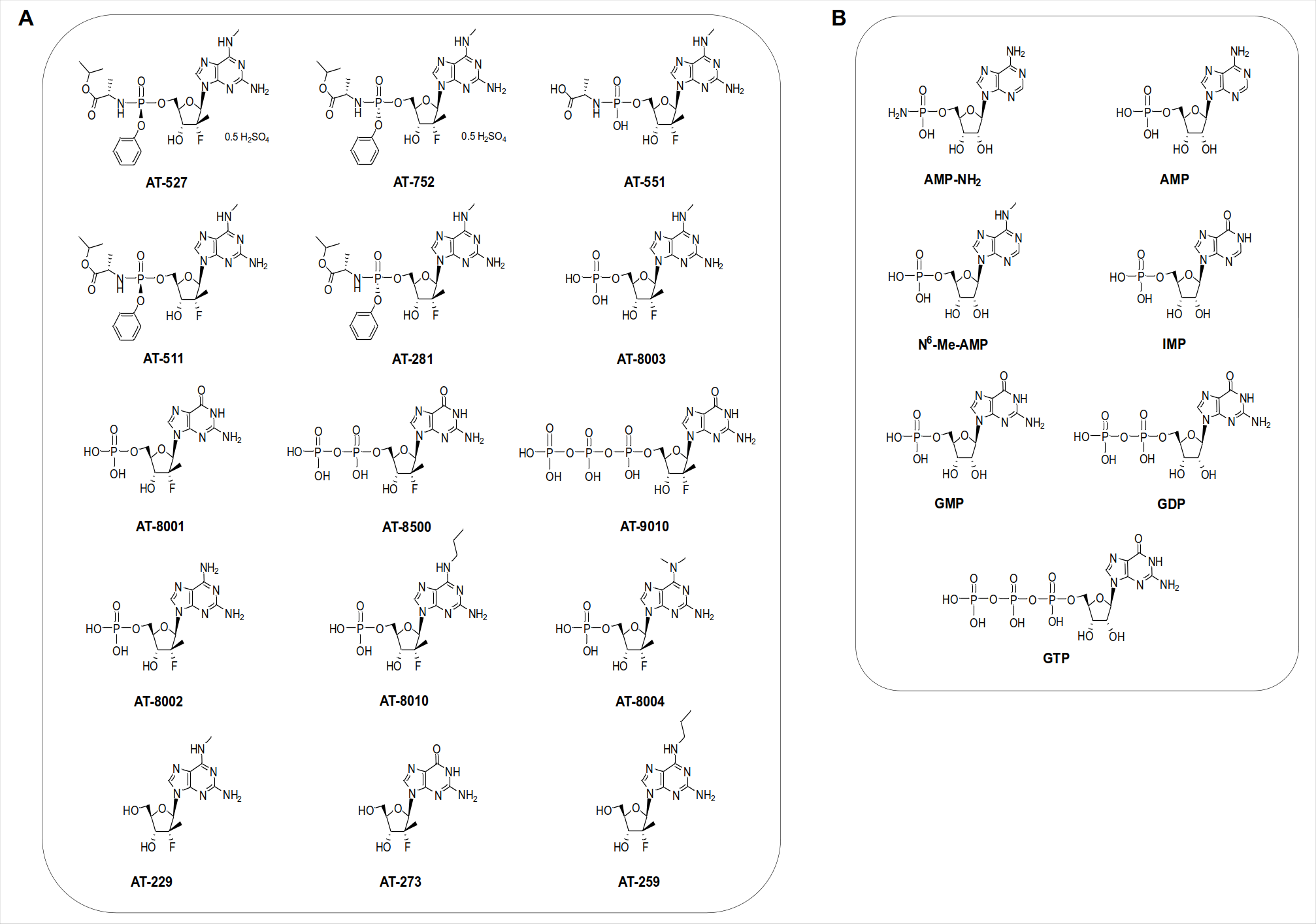

Supplement: S3 Fig — Structure of (A) AT-compounds and (B) reference compounds mentioned in the manuscript. (TIF) [file pbio.3002743.s003.tif]

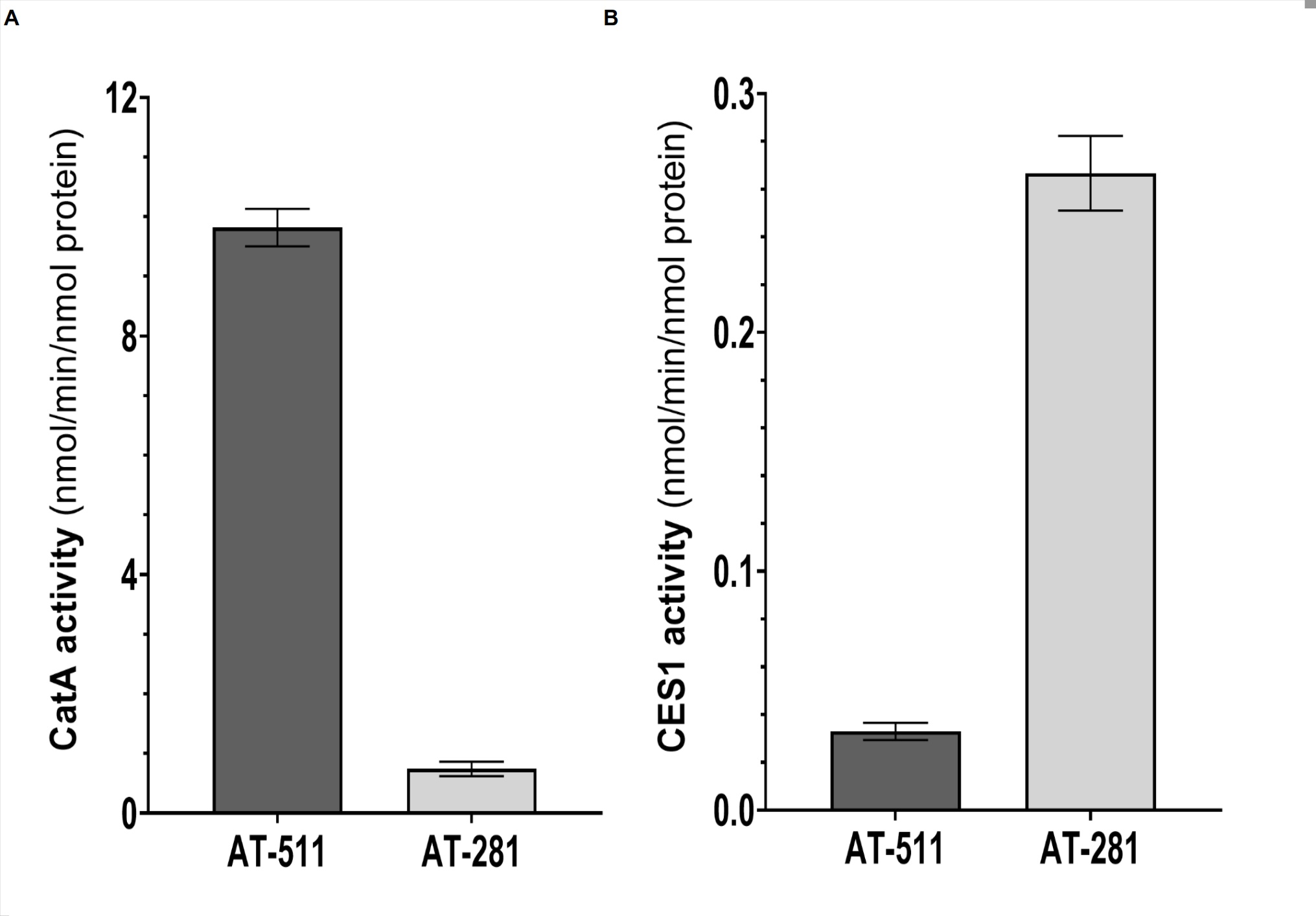

Supplement: S4 Fig — Activity of CatA (panel A) or CES1 (panel B) with either AT-511 (SP isomer) or AT-281 (RP isomer) as substrates. Approximately 20 nM CatA was incubated 45 min at 37°C with 100 μM substrate. About 100 nM CES1 was incubated 2 h at 37°C with 100 μM substrate. Bars show mean values (± SD) of 3 independent experiments. The data underlying this supporting figure can be found at https://zenodo.org/records/12606239. (TIF) [file pbio.3002743.s004.tif]

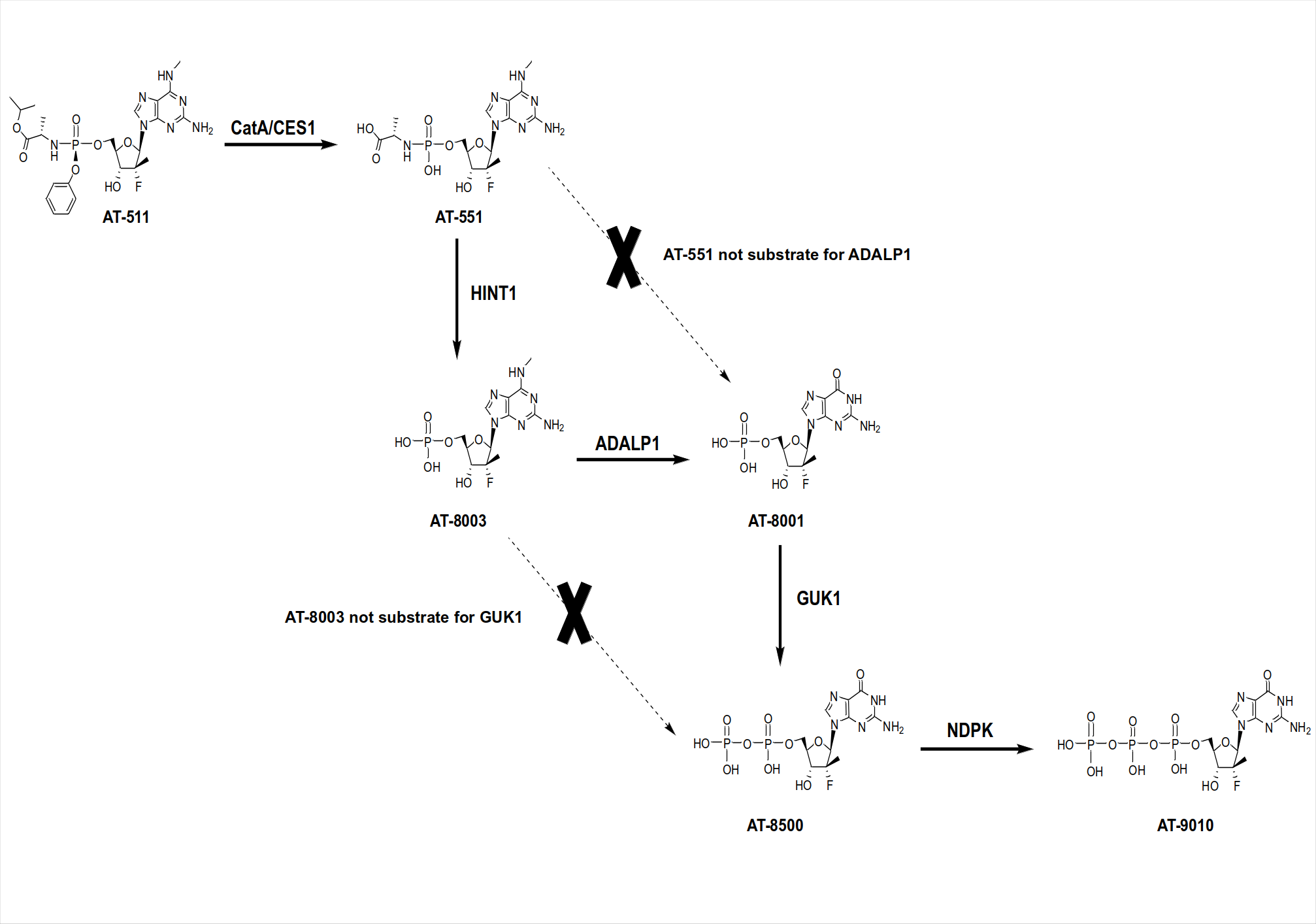

Supplement: S5 Fig — Activation pathway of bemnifosbuvir follows this specific order of reaction. As shown in Table 1, activity assay of ADALP1 with AT-551 as substrate and GUK1 with AT-8003 as substrate did not show any conversion even with 10-fold more enzyme than our standard protocol. (TIF) [file pbio.3002743.s005.tif]

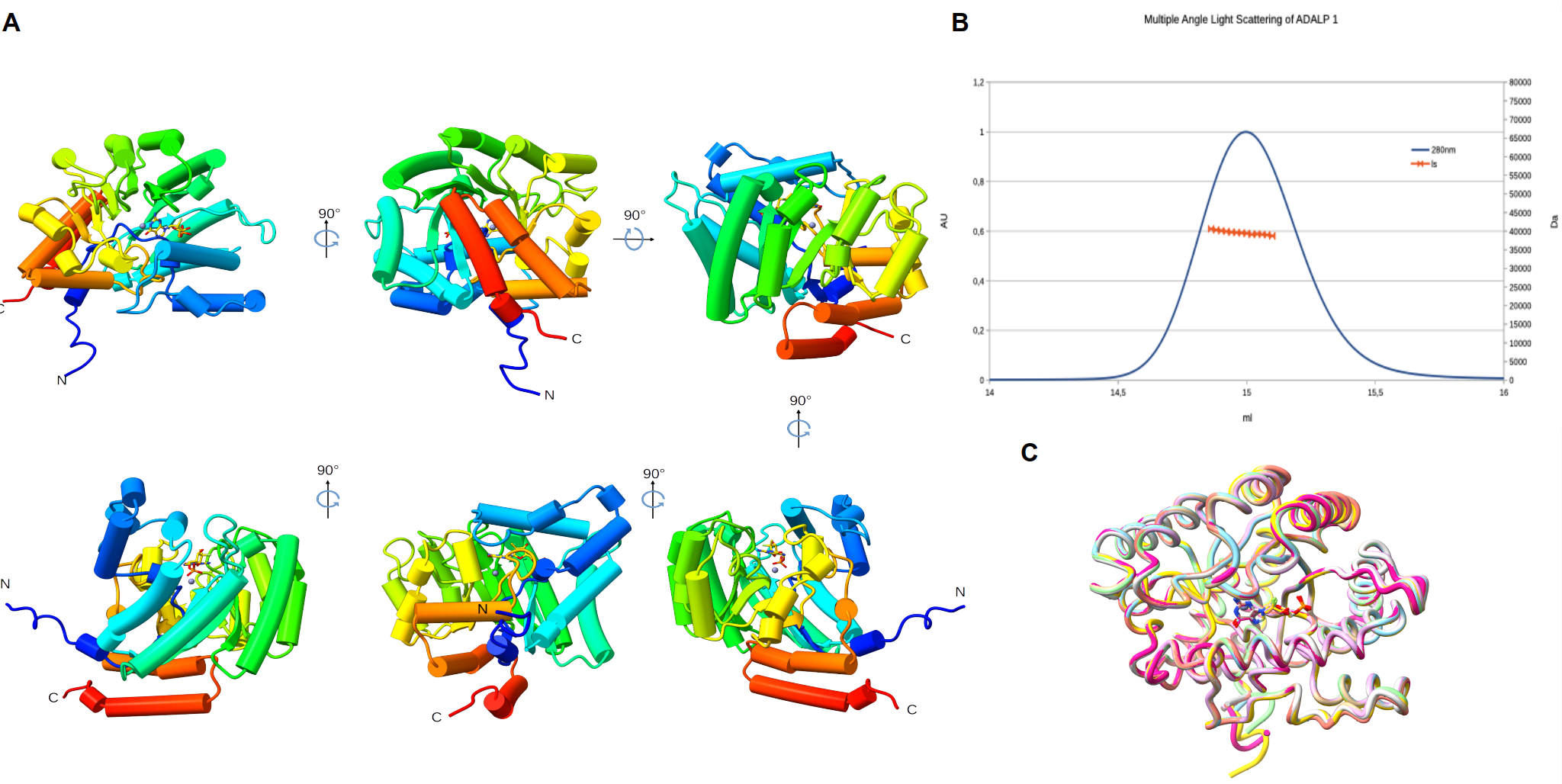

Supplement: S6 Fig — (A) The protein structure is represented in cylinders and stubs and colors in rainbow color code. Different orientations are presented in respect to the previous one. (B) Multiple Angle Light Scattering result graph, presenting the elution curve followed at 280 nm (blue line) and the corresponding scattering graph (orange line), experiment show the homogenity of the sample. (C) The asymmetric unit contained 8 molecules that all contained the compound, presented the superimposition of the 8 chains present showing that they are virtually identical. (TIF) [file pbio.3002743.s006.tif]

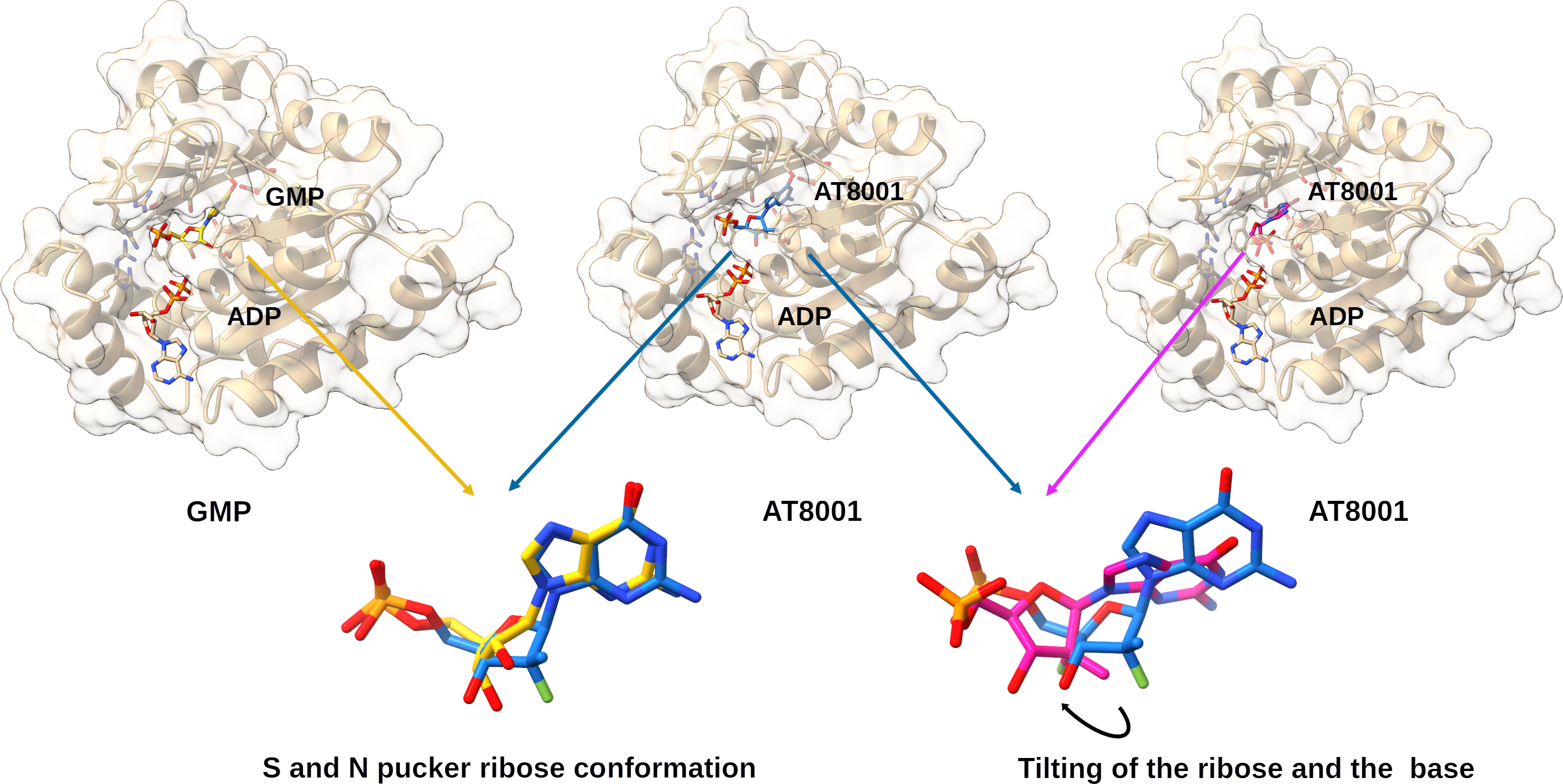

Supplement: S7 Fig — The human GUK1 model is represented in ribbon and transparent surface, while the compound is represented in sticks with heteroatom colors and ADP (brown) at the active site. In the left panel is presented GUK1 in complex with GMP (yellow); in the central and right panel are presented, respectively, GUK1 in complex with AT-8001 in its theoretical position (blue), i.e., superposed with GMP and docked (pink) in the cavity. Below is presented a comparison of the superimposition of the GMP with the theoretical position of AT-8001, highlighting a different ribose pucker conformation, and the superimposition of the theoretical position of AT-8001 with the docked AT-8001, highlighting a tilting of the ribose and the base to reduce steric hindrance between the 2′ methyl with the main chain. (TIF) [file pbio.3002743.s007.tif]

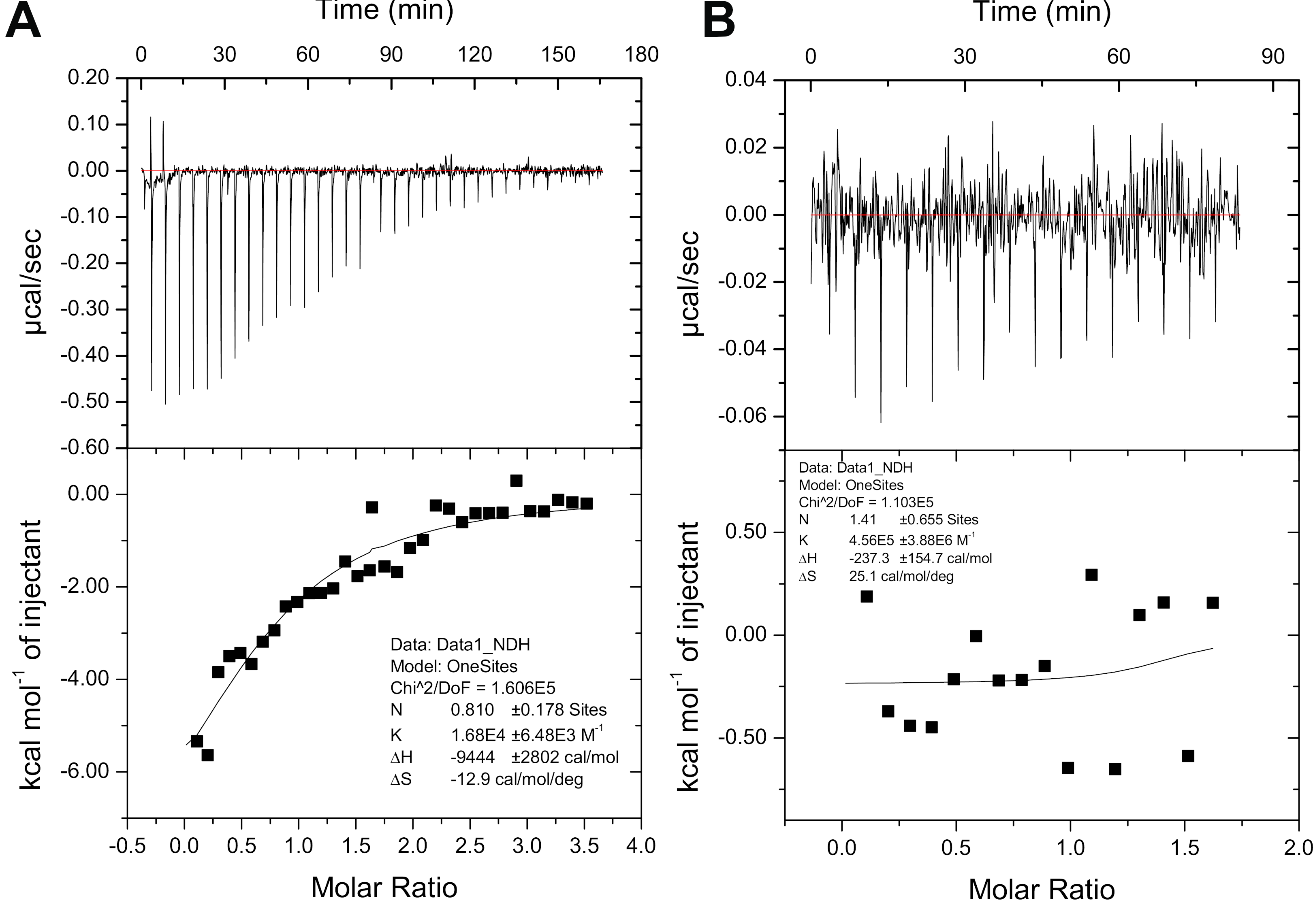

Supplement: S8 Fig — (A) Titration of AMP binding onto HINT1. Top part of the figure shows an exothermic association of AMP and HINT1; bottom part shows the fitting of the data considering 1 site model association, showing Ka approximately 1.7 104 M−1. (B) Titration of AT-8003 binding onto HINT1. The experimental condition fails to report association. Top part of the figure shows absence of peaks defining association of AT-8003 to HINT1; bottom part shows the random fitting of the data considering 1 site model. The data underlying this supporting figure can be found at https://zenodo.org/records/12606239. (TIF) [file pbio.3002743.s008.tif]

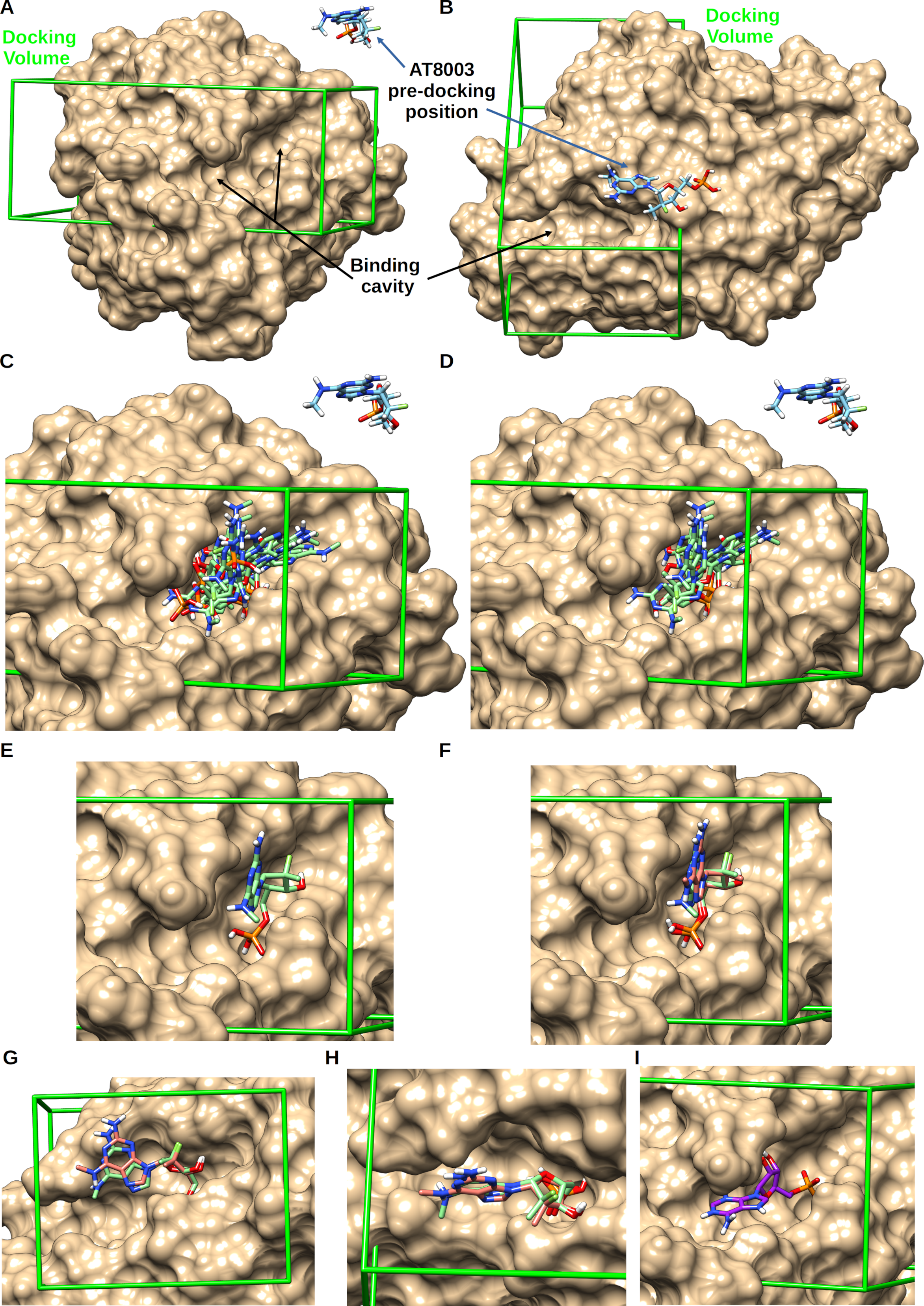

Supplement: S9 Fig — (A, B) Two views (side and tilted) visualizing the criteria for docking experiment: HINT1 structure is presented as the accessible surface (colored wheat). The green box represents the docking grid encompassing the binding and catalytic site; AT-8003 in predocking position (colored in blue) is outside the computational grid and far from the protein target. (C) Docking result overlapping all 10 poses. Docked molecules are colored in light green. None of the molecules are binding following the binding cleft. (D) Similar to C except only remains the 6 poses for which the phosphate is oriented toward the catalytic site. (E) The one pose that is similar to the experimental data. (F, G, H) Superimposition of the docked AT-8003 with the AT-8003 (colored in light pink) measured by crystallography. Phosphate, ribose, and part of the base are fully superimposed, and only the extremity of the base is shifted. (I) Control of docked AMP (colored in purple) showing that the molecule is fully positioned within the binding site. (TIF) [file pbio.3002743.s009.tif]
