# Supplementary material for: The activation cascade of the broad-spectrum antiviral bemnifosbuvir characterized at atomic resolution
Source: PLoS Biol. 2024 Aug 27;22(8):e3002743. doi: 10.1371/journal.pbio.3002743 (PMC11349198; doi:10.1371/journal.pbio.3002743)

# Raw data of SDS-PAGE gels

The SDS-PAGE gels 1 to 5 presented below were used to generate S2 figure panel. The image of the gel used to check the purity of NDPK protein after gel filtration (gel n°5) was not of sufficient quality to visualize the molecular weight marker. Thus, a complementary gel is presented here as supplementary data to confirm the size of NDPK protein obtained, corresponding to our expectations.

List of the following gels:

1- Purification steps of HINT1
2- Purification steps of ADALP1
3- Purification steps of GUK1
4- IMAC purification steps of NDPK
5- GF purification step of NDPK
6- Control of NDPK

The following legend was used to identify samples of the gels:

Lys = lysate
FT = flow through
W = wash
E = elution
Cleavage = fractions from his-tag cleavage steps
GF = gel filtration fractions
X = lane not presented in the final figure/not related to the experiments

The gels were stained with Coomassie blue and images were captured thanks to the Gel Doc™ EZ System with a White Light Sample Tray (Bio-Rad), and the Image Lab™ software (Bio-Rad).

Gel n°1

HINT1

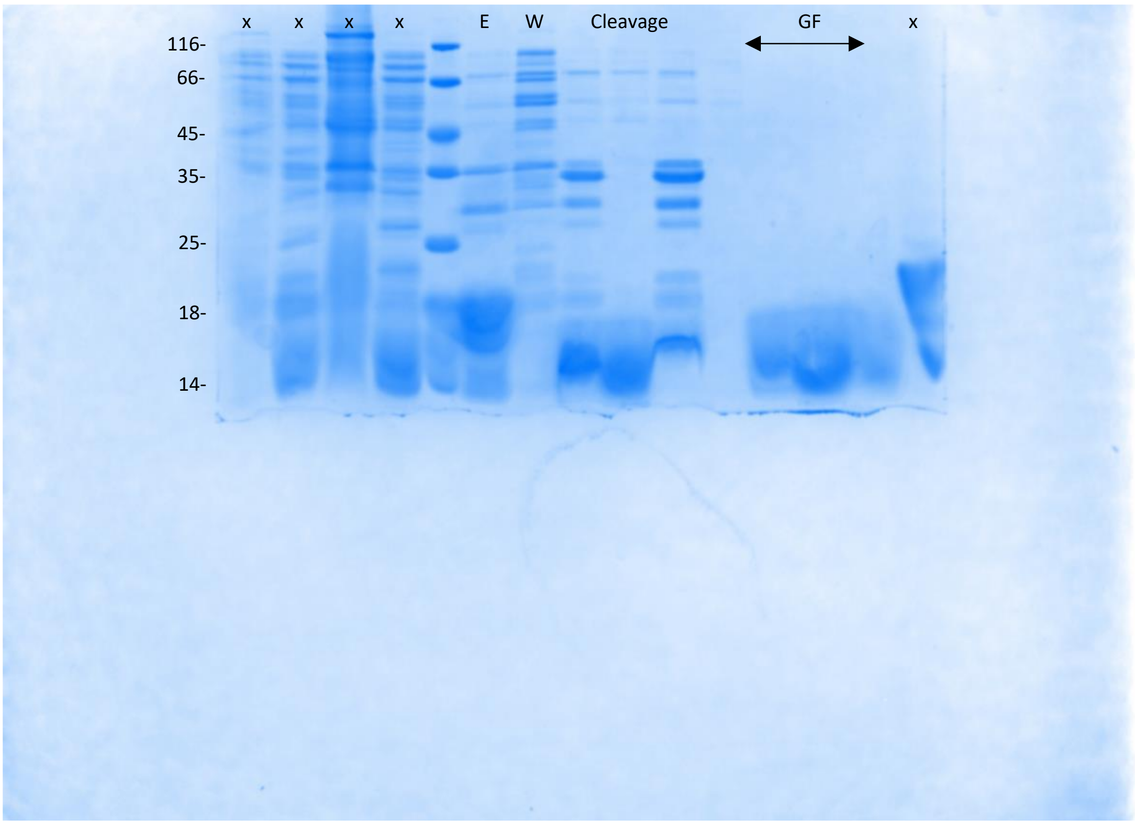

Gel n°2

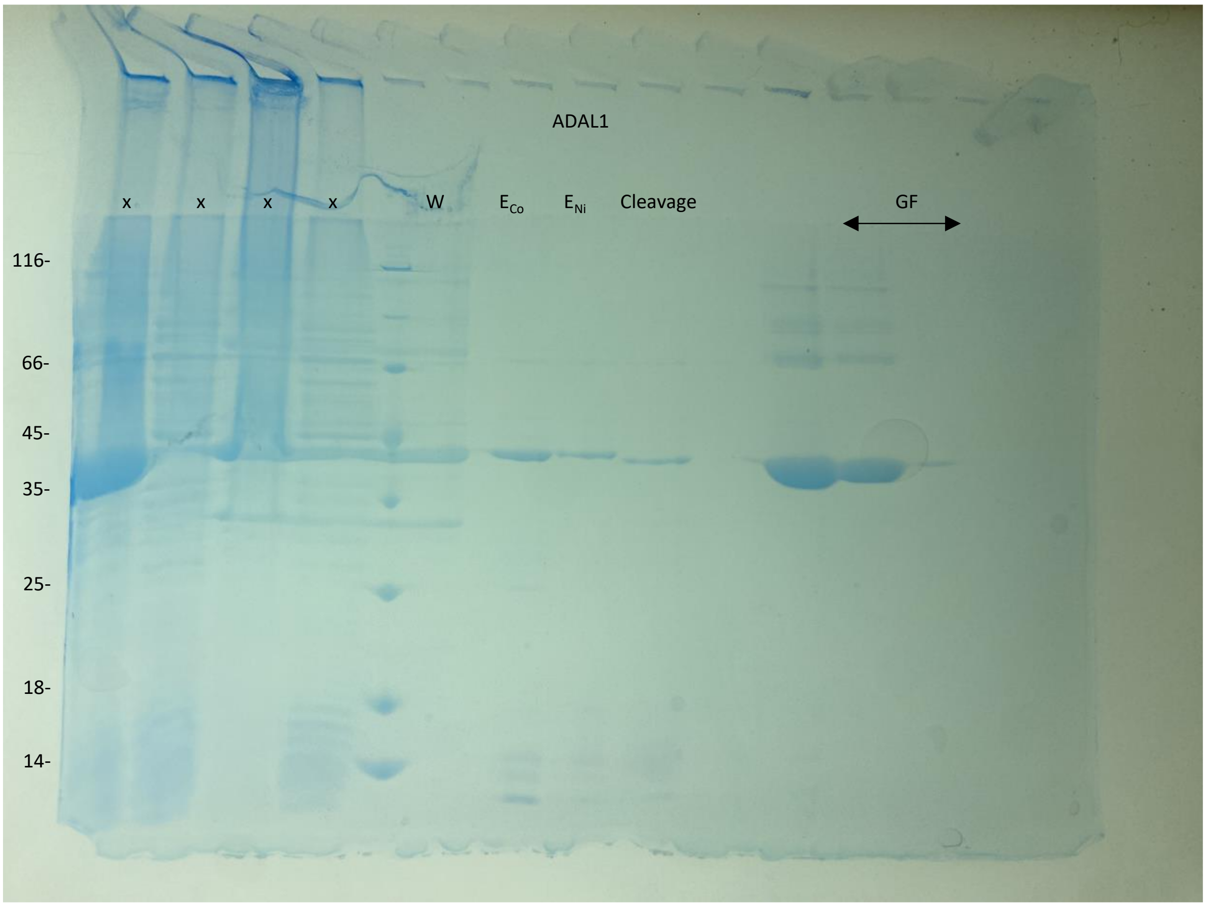

Gel n°3

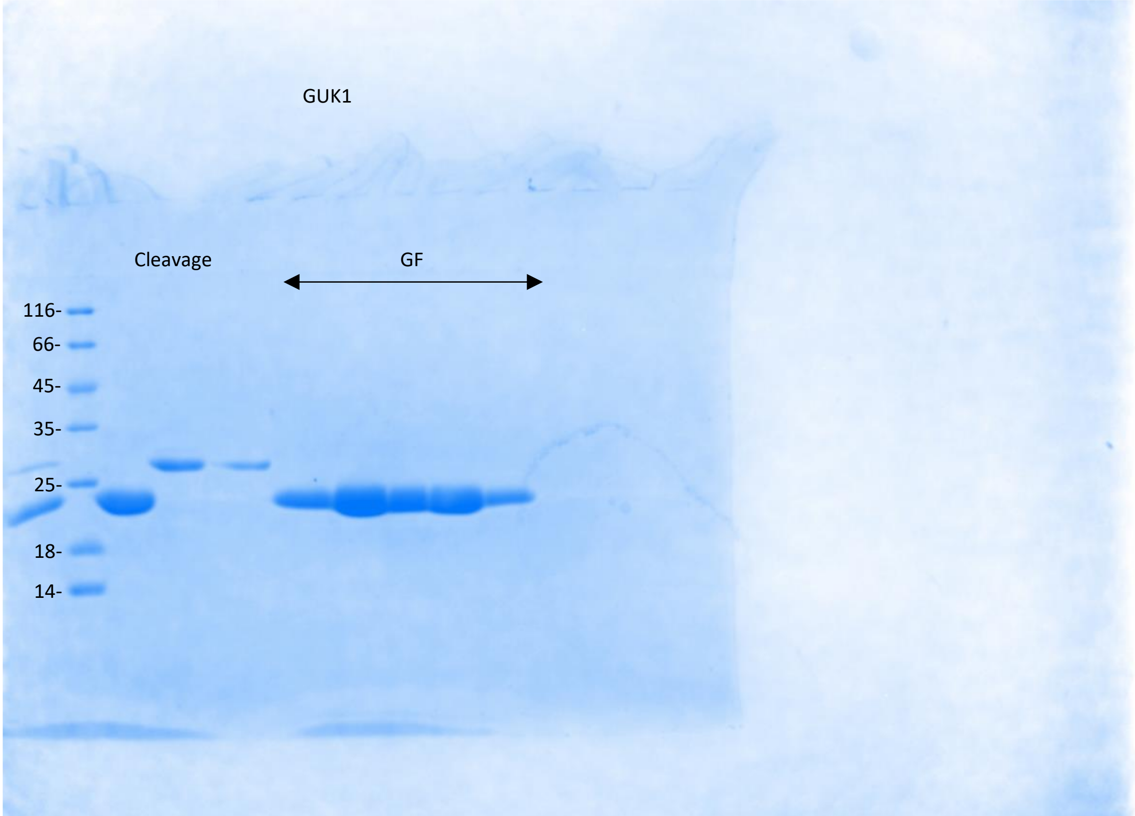

Gel n°4

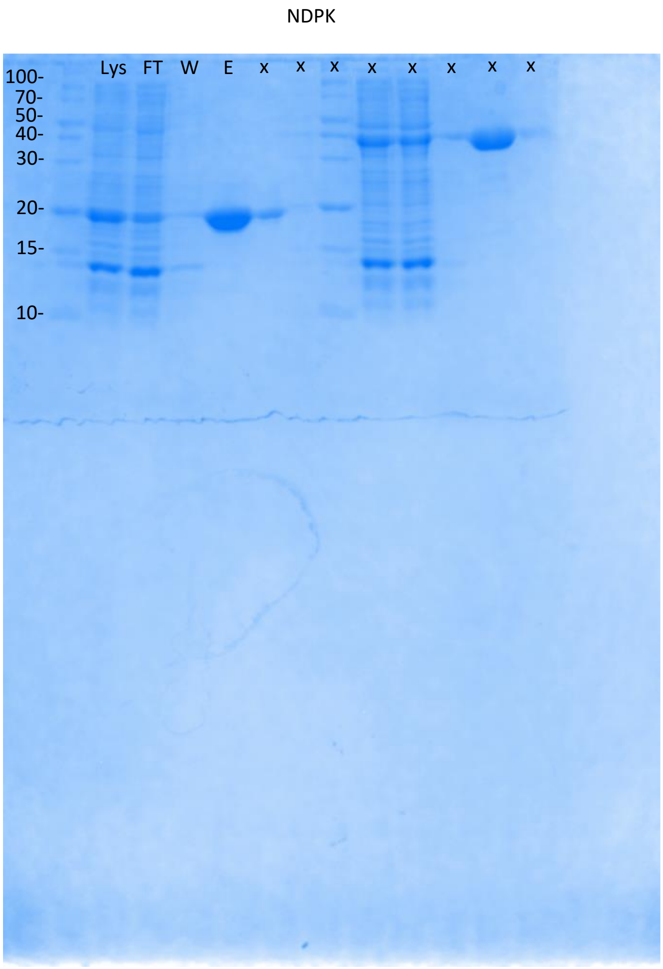

Gel n°5

NDPK

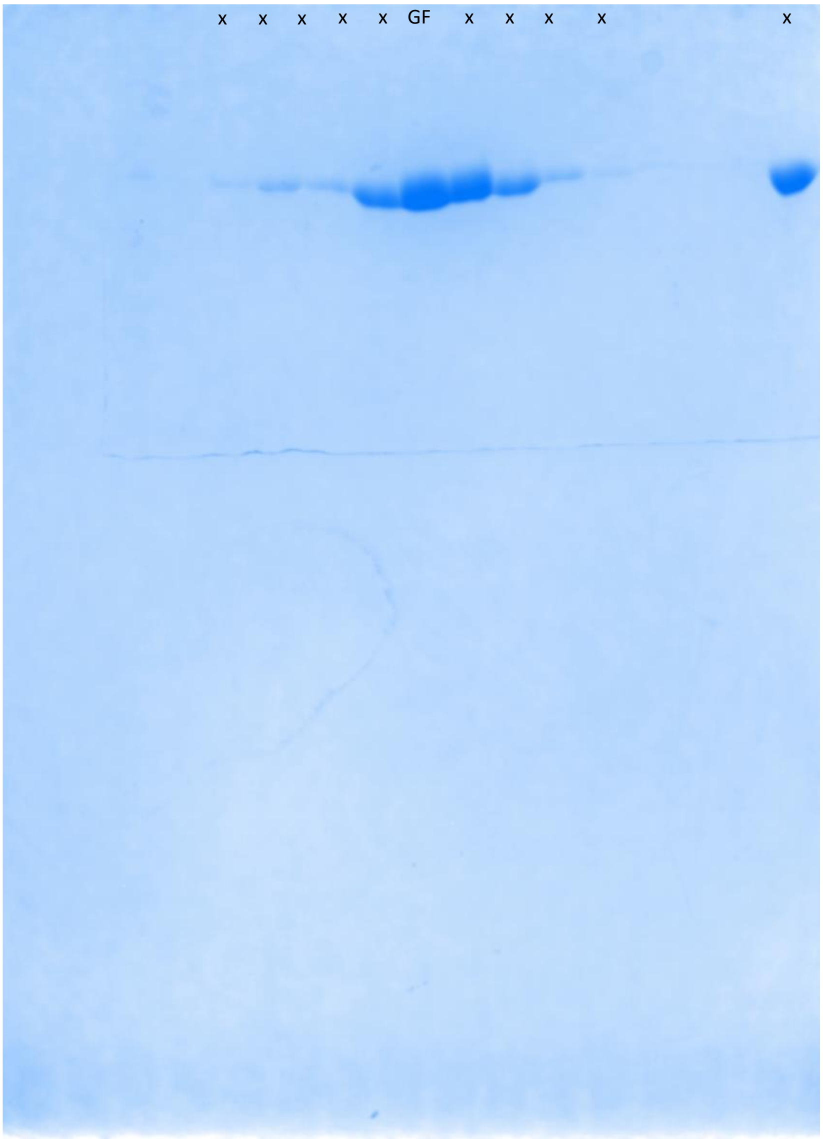

Gel n°6

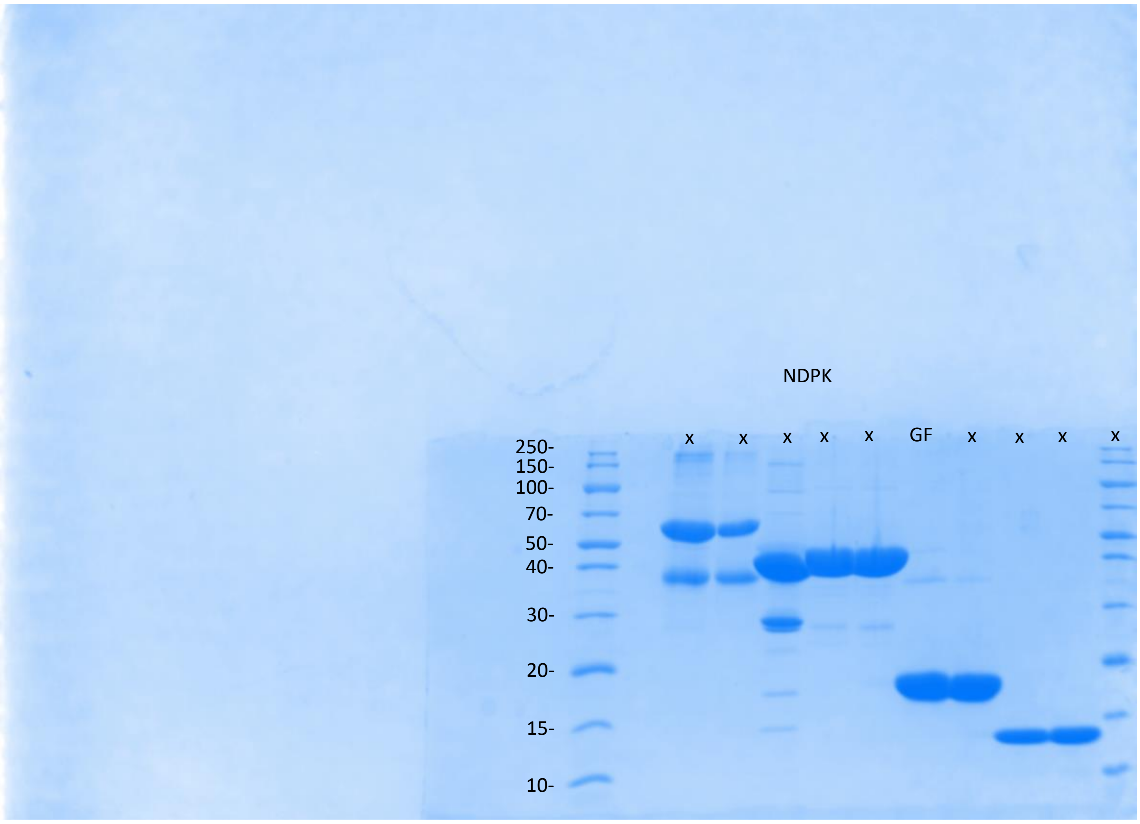

Supplement: S1 Raw Images — (PDF) [file pbio.3002743.s010.pdf]
